# Phase 2, randomized, double‐blind, placebo‐controlled, 4‐week study to evaluate the safety and efficacy of OPA‐ 15406 (difamilast), a new topical selective phosphodiesterase type‐4 inhibitor, in Japanese pediatric patients aged 2–14 years with atopic dermatitis 

**DOI:** 10.1111/1346-8138.15137

**Published:** 2019-11-11

**Authors:** Hidehisa Saeki, Naoko Baba, Kazuhide Oshiden, Yuji Abe, Hidetsugu Tsubouchi

**Affiliations:** ^1^ Department of Dermatology Nippon Medical School Tokyo Japan; ^2^ Kanagawa Children’s Medical Center Kanagawa Japan; ^3^ Headquarters of Clinical Development Otsuka Pharmaceutical Co., Ltd. Osaka Japan; ^4^ Headquarters of Clinical Development Otsuka Pharmaceutical Co., Ltd. Tokyo Japan; ^5^ Medical Affairs Otsuka Pharmaceutical Co., Ltd. Osaka Japan

**Keywords:** atopic dermatitis, difamilast, OPA‐15406, pediatric patients, phosphodiesterase type‐4 inhibitor

## Abstract

The safety and efficacy of OPA‐15406 (international non‐proprietary name, difamilast; also referred to as MM36), a new topical, selective phosphodiesterase type‐4 inhibitor, in Japanese pediatric patients with atopic dermatitis aged 2–14 years were evaluated in a phase 2, randomized, double‐blind, vehicle‐controlled, 4‐week study. Seventy‐three patients were randomized 1:1:1 to receive OPA‐15406 0.3%, OPA‐15406 1% or vehicle ointment twice daily for 4 weeks. The mean age of patients was similar across treatment groups. No deaths or serious treatment‐emergent adverse events were reported; all treatment‐emergent adverse events were mild or moderate in severity. The incidence of treatment‐emergent adverse events leading to treatment discontinuation was 4.2% (1/24) in the OPA‐15406 0.3% group, 4.0% (1/25) in the OPA‐15406 1% group and 16.7% (4/24) in the vehicle group, all of which were worsening of atopic dermatitis. Both OPA‐15406 groups demonstrated a higher incidence of success in the Investigator Global Assessment score compared with the vehicle group over the 4‐week study. The OPA‐15406 groups also showed greater improvements from baseline compared with the vehicle group in the Investigator Global Assessment score, Eczema Area and Severity Index overall score and subscale (erythema, induration/papulation, excoriation and lichenification) scores, Visual Analog Scale pruritus score, Patient‐Oriented Eczema Measure score, and percentage of affected body surface area over the 4‐week study. Topical OPA‐15406 twice daily for 4 weeks was considered a safe and effective treatment option in this phase 2 study in pediatric patients with atopic dermatitis, and phase 3 development is currently ongoing.

## Introduction

Atopic dermatitis (AD) is a common chronic relapsing inflammatory skin disease characterized by dry skin, eczematous lesions and pruritus.^1^, ^2^, ^3^ Pruritus provokes scratching and excoriation, which can lead to further aggravation of inflammation, increased risk of infection, and crusting and lichenification.^4^, ^5^ Patients with AD have an associated burden of sleep disturbance, functional impairment, anxiety, depression, anger and reduced health‐related quality of life (QOL).^6^, ^7^ AD affects 10–20% of children in developed countries.^8^ Approximately 60% of patients develop eruptions in the first year of life, and 90% of patients develop eruptions before the age of 5 years.^4^ The clinical manifestations of AD are categorized into three stages according to age: infancy, childhood and adolescence.^9^, ^10^ Although the morphology of AD lesions varies depending on the age of patients, a hallmark of AD, in all stages, is pruritus.^11^ AD and the associated pruritus may impact childhood growth due to impaired growth hormone release caused by sleep disturbance.^12^, ^13^ Although AD is not always recognized as a serious medical condition by health‐care professionals, it can have a significant negative impact on QOL. Children experience social embarrassment secondary to visible lesions, which also impacts their psychosocial well‐being.^3^ Both QOL and psychosocial well‐being are known to negatively correlate with itch severity.^14^

The complexity of AD is associated with a multifactorial pathogenesis, including genetic and environmental factors, impaired skin barrier and immunological abnormality, which is not yet fully understood.^9^ Currently, there is no cure for AD, but symptoms can be managed through avoidance of environmental and psychological triggers and proper skin care with emollients and moisturizers to maintain skin barrier function. Therefore, the primary goal of treatment is to improve barrier function, suppress inflammation and minimize the risk of infection, which is a frequent complication of AD.^1^, ^2^, ^3^, ^4^, ^5^ Topical therapies play a central role in the treatment of AD. Emollients—which restore skin barrier function—and topical corticosteroids (TCS) and topical calcineurin inhibitors (TCI)—which suppress inflammation—are the most commonly used topical medications.^1^, ^5^

OPA‐15406 (international non‐proprietary name, difamilast; also referred to as MM36)^15^ is a new selective phosphodiesterase type‐4 (PDE4) inhibitor developed by Otsuka Pharmaceutical Co., Ltd (Tokyo, Japan).^16^ PDE4 has been reported to contribute to the pathogenesis of inflammatory disorders, and AD is thought to be caused by elevated PDE4 activity in inflammatory cells.^17^, ^18^ Therefore, PDE4 inhibition is a potential therapeutic target for AD.^1^, ^5^ In a previous phase 2 study in patients with AD aged 10–70 years conducted in Australia, Poland and the USA, OPA‐15406 1% ointment exerted beneficial therapeutic effects, with a low incidence of adverse events (AE).^19^ OPA‐15406 1% ointment also provided favorable efficacy and safety profiles in a phase 2 study in Japanese patients with AD aged 15–70 years.^20^ Furthermore, in a maximal‐use phase 2 study in pediatric patients aged 2–17 years conducted in the USA, OPA‐15406 1% ointment demonstrated therapeutic benefits, was minimally absorbed, and was safe and well tolerated under maximal‐use conditions.^15^ Subsequently, the objective of this study was to evaluate the safety and efficacy of topical OPA‐15406 in Japanese pediatric patients with AD aged 2–14 years.

## Methods

This study was conducted in accordance with the provisions of the Declaration of Helsinki, the International Conference on Harmonisation Good Clinical Practice Consolidated Guideline, and the applicable local laws and regulatory requirements in Japan. The protocol was approved by the institutional review board for each study site. Written informed consent was obtained from the patients’ legal guardians instead of the patients before participation in the study. If possible, assent (consent not bound by the legal restrictions obtained from pediatric patients) was obtained from patients.

### Patients

Male and female outpatients aged 2–14 years who were diagnosed with AD according to the Hanifin and Rajka criteria^21^ and had an Investigator Global Assessment (IGA) score^22^ of 2 (mild) or 3 (moderate) at baseline were eligible for inclusion in this study. Patients were required to have 5–40% of their body surface area (BSA)^23^ affected by AD.

Patients with a clinically significant complication or history of any disorder, judged by the investigator to prevent safety and efficacy assessments, and those with clinically significant abnormal laboratory test values, blood pressure and pulse rate results, or 12‐lead electrocardiogram (ECG) findings were excluded from the study. Patients who could not stop using TCS, immunosuppressors (such as TCI), retinoids or antihistamines from 7 days prior to the baseline examination; those who could not stop using systemic corticosteroids, immunosuppressors, antimetabolites, retinoids or biologics from 28 days prior to the baseline examination; those who could not stop using ultraviolet phototherapy from 28 days prior to the baseline examination; or those who could not continue the study without changing the dose of systemic antihistamines, sodium cromoglicate, tranilast or suplatast tosilate from 7 days prior to the baseline examination were also excluded. Female patients aged 7–14 years who were pregnant or suspected of being pregnant were also excluded.

### Study design

**Figure 1:**
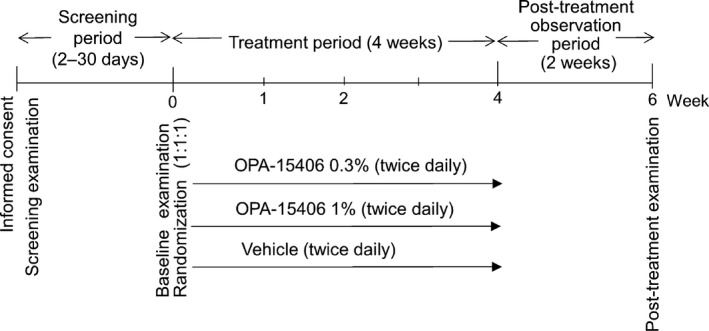
Study design to evaluate the safety and efficacy of topical OPA‐15406 in pediatric patients aged 2–14 years with atopic dermatitis.

This phase 2, randomized, double‐blind, vehicle‐controlled study was conducted at eight study sites in Japan between January 2017 and June 2017 (http://ClinicalTrials.gov identifier: NCT03018691). The sample size of 20 patients for each group was established in consideration of the feasibility of the study. The study consisted of a screening period (2–30 days), a treatment period (4 weeks) and a post‐treatment observation period (2 weeks). The screening period was defined as the period between the day of the screening examination and the day of the baseline examination. Patients meeting the inclusion criteria and not meeting any of the exclusion criteria at the baseline examination entered the treatment period, which was defined as the period between the day of the baseline examination and the day of the week 4 examination (or the day of discontinuation; Fig. 1).

Patients were allowed to discontinue study participation at any time for any reason without any medical disadvantage. If a patient discontinued participation during the treatment period, the examination at discontinuation was conducted.

Eligible patients were randomized in a 1:1:1 ratio to one of the three investigational medical product (IMP) treatment groups using a dynamic allocation method: OPA‐15406 0.3% (w/w), OPA‐15406 1% (w/w) or vehicle ointment. Vehicle was employed as placebo in this study. Randomized patients received the respective IMP twice daily approximately 12 h apart between the morning and night applications for 4 weeks. The IMP allocation manager prepared a randomization table to set the interactive web response system. The appearance of the vehicle ointment was identical to that of the OPA‐15406 ointments.

The investigator instructed the patient and the patient’s legal guardian to apply the IMP to the treatment area. The treatment area was equivalent to the affected BSA at baseline. The expanded affected BSA or the newly observed affected BSA after the baseline examination was also included in the treatment area. Even when the affected BSA was relieved, IMP application on that area was to be continued as the treatment area. The patient’s total BSA was calculated based on the height and bodyweight at the screening examination according to Mosteller’s formula.^23^ The formula for calculation of the dose for each patient was as follows: total BSA (m^2^) × percentage of treatment area (%) × 10 g/m^2^.

### Safety and efficacy assessments

Safety assessments included observation of treatment‐emergent adverse events (TEAE), clinical laboratory tests (hematology, serum chemistry and qualitative urinalysis), physical examinations, vital sign assessments (blood pressure, pulse rate, body temperature and bodyweight) and 12‐lead ECG examination. The severity of TEAE was classified as mild, moderate or severe. The causal relationship between a TEAE and the IMP was assessed as “not related” or “related”.

Efficacy assessments included the incidence of success in the IGA score at weeks 1, 2 and 4. The incidence of success in the IGA score was defined as the percentage of patients who achieved a score of 0 or 1 with an at least 2‐grade reduction from baseline. The IGA score was assessed by using a 5‐point scale (0, clear; 1, almost clear; 2, mild; 3, moderate; 4, severe/very severe).^22^ Other efficacy assessments included the changes from baseline at weeks 1, 2 and 4 in the: (i) IGA score; (ii) Eczema Area and Severity Index (EASI) overall score and subscale (erythema, induration/papulation, excoriation and lichenification) scores;^24^ (iii) Visual Analog Scale (VAS) pruritus score (only for patients aged 7–14 years);^25^ and (iv) Patient‐Oriented Eczema Measure (POEM) score.^26^ Furthermore, the change from baseline at weeks 1, 2 and 4 in the percentage of affected BSA was also assessed.

### Pharmacokinetics

Pharmacokinetic (PK) assessments included the mean plasma trough concentration (ng/mL) and the mean normalized plasma trough concentration by dose derived from the percentage of affected BSA (ng/mL/mg) of OPA‐15406 at weeks 1 and 4. On the day of measurement, the patient visited the study site without morning application of the IMP. The plasma concentration of OPA‐15406 was determined by using liquid chromatography with tandem mass spectrometry at LSI Medience Corporation (Tokyo, Japan).

### Statistical analysis

The safety dataset included all patients who received the IMP at least once. The efficacy dataset consisted of all patients who received the IMP at least once and had assessment data after the initiation of the IMP application.

Treatment‐emergent adverse events were coded to preferred terms according to the Medical Dictionary for Regulatory Activities (MedDRA)/J version 20.0. The number and percentage of patients who experienced TEAE were calculated by treatment group. TEAE by severity, TEAE causally related to the IMP, TEAE resulting in death, serious TEAE and TEAE leading to treatment discontinuation were summarized for each treatment group.

For the incidence of success in the IGA score at weeks 1, 2 and 4, the incidence of success for each treatment group and its 95% confidence interval (CI) were calculated, along with the difference between each OPA‐15406 group and the vehicle group. The primary analysis included the difference in the incidence of success adjusted by the severity of baseline IGA score and its 95% CI using the Cochran–Mantel–Haenszel method. The Cochran–Mantel–Haenszel method was performed using baseline IGA score (mild or moderate) as a stratification factor, and the *P*‐value was calculated. No multiplicity adjustment was performed.

Patients achieving success in the IGA score were regarded as responders. Patients with missing IGA data were counted as non‐responders for that visit.

A mixed‐model repeated measures analysis was applied for the change from baseline in the IGA score at weeks 1, 2 and 4, with factors of treatment (OPA‐15406 0.3% or 1% group and vehicle group), time point, baseline IGA score (mild or moderate), and interaction between treatment and time point. The least square (LS) mean was calculated by treatment group and by examination time point. The differences in the LS means between each OPA‐15406 group and the vehicle group, and the 95% CI and *P*‐value were calculated. The other efficacy assessments were analyzed in the same manner as the change in IGA score from baseline.

## Results

### Patients

**Figure 2:**
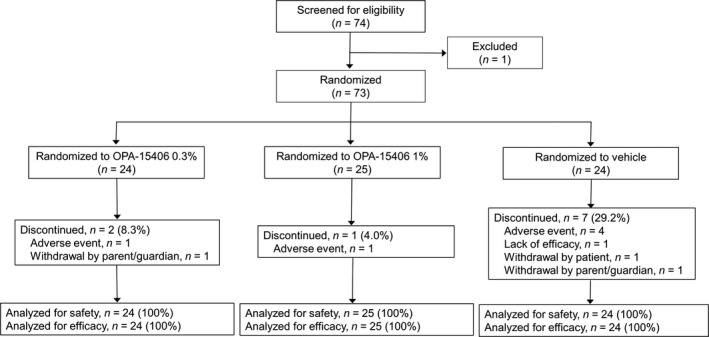
Patient disposition.

Of 74 patients screened, 73 patients were randomized to one of the three treatment groups: OPA‐15406 0.3% group (*n* = 24), OPA‐15406 1% group (*n* = 25) or vehicle group (*n* = 24). In total, 63 patients (86.3%) completed the study. The completion rates in the OPA‐15406 0.3%, OPA‐15406 1% and vehicle groups were 91.7% (22/24), 96.0% (24/25) and 70.8% (17/24), respectively (Fig. 2). The most frequently reported reasons for discontinuation in all treatment groups were AE (8.2%), followed by withdrawal by parent/guardian (2.7%).

Demographic and clinical characteristics of the patients are presented in Table 1. The majority of patients were male (71.2% [52/73]), and the overall mean ± standard deviation (SD) age was 8.3 ± 3.4 years. The mean age at baseline in the OPA‐15406 0.3%, OPA‐15406 1% and vehicle groups was 8.5 ± 3.8, 7.9 ± 3.5 and 8.5 ± 3.1 years, respectively, showing that the mean age at baseline was similar across the treatment groups. In total, 12 patients (16.4%) had an IGA score of 2, and 61 (83.6%) had an IGA score of 3.

**Table 1:** Demographic and clinical characteristics

	OPA‐15406 0.3%, *n* = 24	OPA‐15406 1%, *n* = 25	Vehicle, *n* = 24
Age, years, mean ± SD	8.5 ± 3.8	7.9 ± 3.5	8.5 ± 3.1
Male, *n* (%)	18 (75.0)	15 (60.0)	19 (79.2)
Weight, kg, mean ± SD	32.4 ± 16.2	28.1 ± 12.4	30.8 ± 13.3
Height, cm, mean ± SD	129.9 ± 24.1	125.8 ± 24.1	130.1 ± 20.6
BMI, kg/m^2^, mean ± SD	18.0 ± 3.7	16.9 ± 2.1	17.2 ± 2.5
AD duration, years, mean ± SD	7.5 ± 3.9	7.3 ± 3.5	7.2 ± 3.3
IGA score, *n* (%)			
Mild disease	4 (16.7)	5 (20.0)	3 (12.5)
Moderate disease	20 (83.3)	20 (80.0)	21 (87.5)
Affected body surface area, *n* (%)			
≥5% to <10%	2 (8.3)	6 (24.0)	3 (12.5)
≥10% to <30%	18 (75.0)	17 (68.0)	18 (75.0)
≥30%	4 (16.7)	2 (8.0)	3 (12.5)

AD duration is the years since onset of AD. AD, atopic dermatitis; BMI, body mass index; IGA, Investigator Global Assessment; SD, standard deviation.

### Safety assessments

Of the 73 patients included in this study, 37 patients (50.7%) experienced TEAE. The incidences of TEAE in the OPA‐15406 0.3%, OPA‐15406 1% and vehicle groups were 45.8% (11/24), 56.0% (14/25) and 50.0% (12/24), respectively.

Treatment‐emergent adverse events observed in at least 5% of patients in any treatment group were worsening of AD (8.3% [2/24]) and influenza (8.3% [2/24]) in the OPA‐15406 0.3% group; upper respiratory tract inflammation (24.0% [6/25]) and blood alkaline phosphatase increased (8.0% [2/25]) in the OPA‐15406 1% group; and worsening of AD (16.7% [4/24]), viral upper respiratory tract infection (8.3% [2/24]) and upper respiratory tract inflammation (8.3% [2/24]) in the vehicle group (Table 2).

**Table 2:** Summary of treatment‐emergent adverse events observed in at least 5% of patients in any treatment group

	OPA‐15406 0.3%, *n* = 24	OPA‐15406 1%, *n* = 25	Vehicle, *n* = 24
Infections and infestations, *n* (%)			
Influenza	2 (8.3)	1 (4.0)	0 (0.0)
Viral upper respiratory tract infection	1 (4.2)	0 (0.0)	2 (8.3)
Investigations, *n* (%)			
Blood alkaline phosphatase increased	0 (0.0)	2 (8.0)	0 (0.0)
Respiratory, thoracic and mediastinal disorders, *n* (%)			
Upper respiratory tract inflammation	1 (4.2)	6 (24.0)	2 (8.3)
Skin and subcutaneous tissue disorders, *n* (%)			
Dermatitis atopic	2 (8.3)	1 (4.0)	4 (16.7)

Treatment‐emergent adverse events were coded to preferred terms according to the Medical Dictionary for Regulatory Activities (MedDRA)/J version 20.0.

The incidences of IMP‐related TEAE were 4.2% (1/24) in the OPA‐15406 0.3% group, 16.0% (4/25) in the OPA‐15406 1% group and 20.8% (5/24) in the vehicle group. Worsening of AD related to the IMP was observed for one patient (4.2%) in the OPA‐15406 0.3% group, one patient (4.0%) in the OPA‐15406 1% group and three patients (12.5%) in the vehicle group. Folliculitis (4.2% [1/24]) in the OPA‐15406 0.3% group, blood alkaline phosphatase increased (8.0% [2/25]) and protein urine present (4.0% [1/25]) in the OPA‐15406 1% group, and pigmentation disorder (4.2% [1/24]) and pruritus (4.2% [1/24]) in the vehicle group were also judged to be related to the IMP.

Treatment‐emergent adverse events observed at the application sites were folliculitis (4.2% [1/24]) and worsening of AD (8.3% [2/24]) in the OPA‐15406 0.3% group, molluscum contagiosum (4.0% [1/25]) and worsening of AD (4.0% [1/25]) in the OPA‐15406 1% group, and worsening of AD (16.7% [4/24]), pigmentation disorder (4.2% [1/24]) and pruritus (4.2% [1/24]) in the vehicle group.

The incidences of TEAE leading to discontinuation (all of which were worsening of AD) were 4.2% (1/24) in the OPA‐15406 0.3% group, 4.0% (1/25) in the OPA‐15406 1% group and 16.7% (4/24) in the vehicle group.

All TEAE were mild or moderate in severity. No deaths or serious TEAE were observed in this study. Overall, no clinically relevant trend in abnormalities was reported based on the clinical laboratory test results, vital sign assessments and 12‐lead ECG examinations.

### Pharmacokinetics

The mean ± SD plasma trough concentrations were 0.842 ± 0.577 ng/mL at week 1 (*n* = 18) and 0.946 ± 1.16 ng/mL at week 4 (*n* = 20) in the OPA‐15406 0.3% group, and 2.90 ± 2.74 ng/mL at week 1 (*n* = 21) and 2.21 ± 1.81 ng/mL at week 4 (*n* = 22) in the OPA‐15406 1% group. The mean ± SD normalized plasma trough concentrations by dose derived from the percentage of affected BSA were 0.196 ± 0.201 ng/mL/mg at week 1 (*n* = 18) and 0.150 ± 0.111 ng/mL/mg at week 4 (*n* = 20) in the OPA‐15406 0.3% group, and 0.210 ± 0.174 ng/mL/mg at week 1 (*n* = 21) and 0.166 ± 0.148 ng/mL/mg at week 4 (*n* = 22) in the OPA‐15406 1% group.

### Efficacy

**Figure 3:**
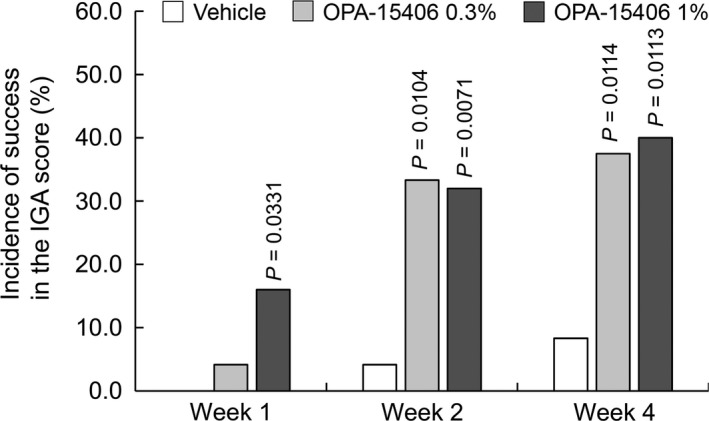
Incidence of success in the IGA score at each examination time point. In total, 24, 24 and 25 patients were examined in the vehicle, OPA‐15406 0.3% and OPA‐15406 1% groups, respectively, at all examination time points. *P*‐values are for the comparison between each OPA‐15406 group and the vehicle group. IGA, Investigator Global Assessment.

The incidences of success in the IGA score at weeks 1, 2 and 4 were 4.17% (95% CI, 0.11–21.12), 33.33% (95% CI, 15.63–55.32) and 37.50% (95% CI, 18.80–59.41), respectively, in the OPA‐15406 0.3% group; 16.00% (95% CI, 4.54–36.08), 32.00% (95% CI, 14.95–53.50) and 40.00% (95% CI, 21.13–61.33), respectively, in the OPA‐15406 1% group; and 0.00% (95% CI, 0.00–14.25), 4.17% (95% CI, 0.11–21.12) and 8.33% (95% CI, 1.03–27.00), respectively, in the vehicle group. The differences between the OPA‐15406 0.3% group and the vehicle group at weeks 1, 2 and 4 were 4.28% (95% CI, ‐3.83–12.40; *P* = 0.3055), 29.49% (95% CI, 9.05–49.93; *P* = 0.0104) and 30.39% (95% CI, 8.51–52.27; *P* = 0.0114), respectively, and those between the OPA‐15406 1% group and the vehicle group were 16.91% (95% CI, 2.19–31.63; *P* = 0.0331), 29.79% (95% CI, 9.91–49.66; *P* = 0.0071) and 31.95% (95% CI, 9.79–54.11; *P* = 0.0113), respectively (Fig. 3). Both OPA‐15406 groups showed a higher incidence of success in the IGA score compared with the vehicle group over the 4‐week study.

The LS mean changes from baseline in the IGA score, EASI score, VAS pruritus score, POEM score and percentage of affected BSA are shown in Table 3. Both OPA‐15406 groups indicated greater improvements from baseline compared with the vehicle group in the IGA score over the 4‐week study. Furthermore, both OPA‐15406 groups showed greater improvements from baseline compared with the vehicle group in the EASI, VAS pruritus and POEM scores over the 4‐week study. It is notable that both OPA‐15406 groups showed a consistent improvement over the 4‐week study in the VAS pruritus score, which is one of the most commonly used methods to assess pruritus severity.^25^

**Table 3:** Summary of least square mean changes from baseline in efficacy parameters

	Baseline		Change from baseline								
			Week 1			Week 2			Week 4		
	Mean (SD)	*n*	LS mean (SE)	*n*	*P*	LS mean (SE)	*n*	*P*	LS mean (SE)	*n*	*P*
IGA											
OPA‐15406 0.3%	2.8 (0.4)	24	−0.50 (0.14)	24	0.0483	−1.13 (0.19)	24	0.0002	−1.04 (0.22)	23	0.0048
OPA‐15406 1%	2.8 (0.4)	25	−0.70 (0.14)	25	0.0033	−1.04 (0.19)	24	0.0006	−1.16 (0.21)	24	0.0014
Vehicle	2.9 (0.3)	24	−0.10 (0.14)	24		−0.06 (0.20)	21		−0.12 (0.23)	18	
EASI											
OPA‐15406 0.3%	12.07 (6.79)	24	−4.61 (0.93)	24	0.0023	−6.68 (1.10)	24	<0.0001	−5.33 (1.28)	23	0.0027
OPA‐15406 1%	9.37 (5.80)	25	−2.99 (0.91)	25	0.0539	−5.06 (1.09)	24	0.0005	−5.52 (1.26)	24	0.0019
Vehicle	10.31 (5.92)	24	−0.45 (0.92)	24		0.66 (1.13)	21		0.56 (1.36)	18	
VAS pruritus											
OPA‐15406 0.3%	54.8 (17.9)	17	−18.61 (6.50)	17	0.0447	−16.79 (5.94)	17	0.2564	−18.00 (7.27)	16	0.0167
OPA‐15406 1%	45.6 (29.0)	17	−12.83 (6.47)	17	0.1557	−24.27 (6.01)	16	0.0488	−17.21 (7.29)	16	0.0198
Vehicle	46.8 (28.9)	17	0.34 (6.46)	17		−6.95 (6.11)	15		8.19 (7.58)	14	
POEM											
OPA‐15406 0.3%	11.4 (5.2)	24	−4.50 (0.82)	24	<0.0001	−5.67 (0.97)	24	<0.0001	−4.33 (1.16)	23	0.0003
OPA‐15406 1%	11.2 (5.0)	25	−4.94 (0.81)	25	<0.0001	−5.13 (0.96)	24	<0.0001	−4.37 (1.14)	24	0.0002
Vehicle	13.0 (7.5)	24	0.36 (0.83)	24		0.98 (1.01)	21		2.26 (1.24)	18	
Affected BSA†											
OPA‐15406 0.3%	21.13 (9.56)	24	−4.08 (1.66)	24	0.1219	−7.72 (1.88)	24	0.0004	−6.06 (2.36)	23	0.0274
OPA‐15406 1%	15.96 (8.60)	25	−4.19 (1.62)	25	0.1036	−6.81 (1.85)	24	0.0010	−8.21 (2.32)	24	0.0050
Vehicle	17.46 (8.65)	24	−0.42 (1.63)	24		2.37 (1.92)	21		1.78 (2.53)	18	

^†^ Data on LS mean change from baseline in the percentage of affected BSA. BSA, body surface area; EASI, Eczema Area and Severity Index; IGA, Investigator Global Assessment; LS, least square; POEM, Patient‐Oriented Eczema Measure; SD, standard deviation; SE, standard error; VAS, Visual Analog Scale.

**Figure 4:**
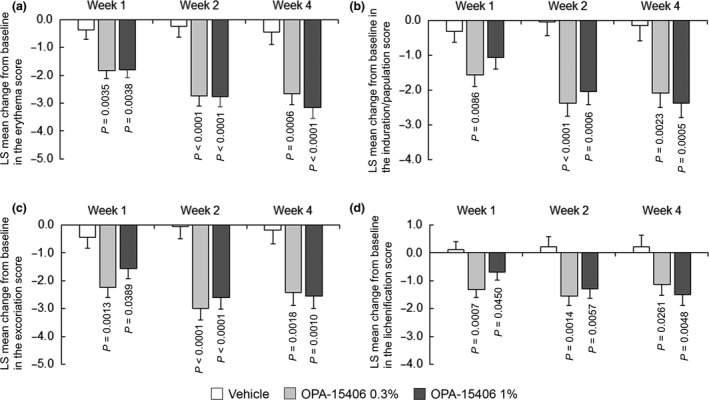
Least square mean change in the Eczema Area and Severity Index subscale scores from baseline at each examination time point: (a) erythema score; (b) induration/papulation score; (c) excoriation score; (d) lichenification score. In the vehicle, OPA‐15406 0.3% and OPA‐15406 1% groups, 24, 24 and 25 patients, respectively, were examined at week 1; 21, 24 and 24 patients, respectively, were examined at week 2; and 18, 23 and 24 patients, respectively, were examined at week 4. *P*‐values are for the comparison between each OPA‐15406 group and the vehicle group. LS, least square.

The LS mean changes from baseline in the subscale EASI (erythema, induration/papulation, excoriation and lichenification) scores are shown in Figure 4. The OPA‐15406 0.3% and 1% groups showed greater improvements from baseline compared with the vehicle group in the subscale scores over the 4‐week study. Furthermore, both OPA‐15406 groups indicated a greater decrease from baseline compared with the vehicle group in the percentage of affected BSA over the 4‐week study (Table 3).

## Discussion

Atopic dermatitis is a common disease in children, which frequently develops in the first years of life and sometimes persists throughout childhood and into adolescence. Pediatric AD imposes an enormous burden on children and their families.^8^ Topical PDE4 inhibitors, including OPA‐15406, have been evaluated for the treatment of patients with AD, and further development is currently ongoing.^27^ This phase 2 study evaluated the safety and efficacy of topical OPA‐15406 in Japanese pediatric patients with AD aged 2–14 years.

In the safety assessment, only one patient each in the OPA‐15406 0.3% and OPA‐15406 1% groups discontinued study participation due to worsening of AD, compared with four patients in the vehicle group. The IMP‐related event of blood alkaline phosphatase increase observed in the OPA‐15406 1% group was consistent with previous reports of benign transient hyperphosphatasemia being occasionally observed in children under the age of 5 years without evidence of bone, gastrointestinal or liver disease on history, physical examination or laboratory investigations.^28^ No deaths or serious TEAE were reported in this study. Overall, the TEAE observed in this study were all mild or moderate in severity. Therefore, topical application of OPA‐15406 in pediatric patients with AD twice daily for up to 4 weeks may provide a favorable safety profile. The results of the safety assessment for topical OPA‐15406 observed in this study are generally consistent with those observed in previous phase 2 studies.^15^, ^19^, ^20^ The mean normalized OPA‐15406 plasma trough concentrations by dose derived from the percentage of affected BSA were similar at weeks 1 and 4, indicating no systemic accumulation following topical dosing.

In the efficacy assessment, both OPA‐15406 groups showed a higher incidence of success in the IGA score relative to the vehicle group. In addition, both OPA‐15406 groups showed greater improvements in the investigator‐reported IGA score, EASI overall score and subscale scores, and patient‐reported VAS pruritus score and POEM score compared with the vehicle group over the 4‐week study. Furthermore, both OPA‐15406 groups showed a greater decrease in the percentage of affected BSA compared with the vehicle group. These findings are clinically meaningful, because the OPA‐15406 groups improved both investigator‐reported and patient‐reported scores while decreasing the percentage of affected BSA. The results of the efficacy assessment observed in this study are generally consistent with those observed in previous phase 2 studies.^15^, ^19^, ^20^ Pruritus, defined as an unpleasant sensation that stimulates a desire to scratch, is the most predominant and uncontrollable symptom of AD.^29^, ^30^ Pruritus is a hallmark of AD, and can lead to skin damage by excoriation and secondary infection, which further aggravates the disease. Nocturnal scratching is a common symptom of AD and can result in sleep disturbance and reduced QOL, especially in children.^30^ Therefore, a new treatment option that can suppress pruritus as well as inflammation is required.

As mentioned above, topical OPA‐15406, a PDE4 inhibitor, is considered to be a safe and effective treatment option for pediatric patients with AD, while relieving pruritus. Our study has some limitations due to the relatively small number of enrolled patients, which was not calculated to be powered for an efficacy assessment based on a statistical standpoint, and the short study period (4 weeks), which was insufficient to evaluate the long‐term safety and efficacy of topical OPA‐15406. Therefore, the currently enrolling phase 3 studies are anticipated to confirm the safety and efficacy of topical OPA‐15406 for pediatric patients with AD (http://ClinicalTrials.gov identifier: NCT03911401 and NCT03961529).

## Conflict of Interest

This study was supported by Otsuka Pharmaceutical Co., Ltd. H. S. was the medical expert for the study and N. B. was the advisor for the study. H. S. and N. B. have received fees for consultation from Otsuka Pharmaceutical Co., Ltd. K. O., Y. A. and H. T. are employees of Otsuka Pharmaceutical Co., Ltd.

## Study Investigators

Takashi Onoduka, M.D. (Asanuma Dermatological Clinic, Hokkaido, Japan), Hidetoshi Takahashi, M.D. (Takagi Dermatological Clinic, Hokkaido, Japan), Hiroshi Sugawara, M.D. (Medical Corporation Bikyukai Kokubu Dermatology, Hokkaido, Japan), Ryuji Maruyama, Ph.D. (Maruyama Dermatology Clinic, Tokyo, Japan), Taizo Hamaguchi, Ph.D. (Medical Corporation H.S.C Hamaguchi Skin Clinic, Tokyo, Japan), Akira Yoshioka, M.D. (Medical Corporation Kojinkai Yoshioka Dermatology Clinic, Osaka, Japan), Ryota Yoshimura, M.D. (Yoshimura Child Clinic, Hyogo, Japan), and Mitsuru Fukazawa, M.D. (Fukazawa Pediatric Clinic, Fukuoka, Japan).
